# Aggregation of poorly crystalline and amorphous components of infectious urinary stones is mediated by bacterial lipopolysaccharide

**DOI:** 10.1038/s41598-019-53359-z

**Published:** 2019-11-19

**Authors:** Jolanta Prywer, Agnieszka Torzewska

**Affiliations:** 1 Institute of Physics, Lodz University of Technology, ul. Wólczańska 219, 90-924 Łódź, Poland; 2 Department of Biology of Bacteria, Faculty of Biology and Environmental Protection, University of Lodz, ul. Banacha 12/16, 90-237 Łódź, Poland

**Keywords:** Bacterial toxins, Bacterial infection, Condensed-matter physics

## Abstract

Poorly crystalline and amorphous precipitate (PCaAP) is one of the components of the so-called infectious urinary stones, which are the result of the activity of urease-producing microorganisms, mainly from the *Proteus* species, in particular *Proteus mirabilis*. The main component of this kind of stones is crystalline struvite (MgNH_4_PO_4_∙6H_2_O). Bacteria can build into the structure of the urinary stone and, in this way, they are one of the components of the urinary stone. From these three components – PCaAP, struvite and *Proteus mirabilis* – PCaAP exhibits the greatest ability to aggregate. The present study focuses on the aggregation of PCaAP. In particular, an influence of lipopolysaccharide (LPS) isolated from *Proteus mirabilis* on aggregation of PCaAP is presented. An aggregation of PCaAP is characterized by cross-sectional area of aggregates and zeta potential. The results demonstrate that, in artificial urine, the influence of freely suspended LPS on aggregation of PCaAP depends on the concentrations of LPS. Small concentrations of freely suspended LPS enhance the aggregation of PCaAP compared to the control test. For high concentrations of freely suspended LPS the formation of aggregates of PCaAP is inhibited. LPS, which is not freely suspended, but covers polystyrene latex beads, has no such properties. The investigations provide evidence for the importance of biological regulation in the PCaAP aggregation process.

## Introduction

**Figure 1:**
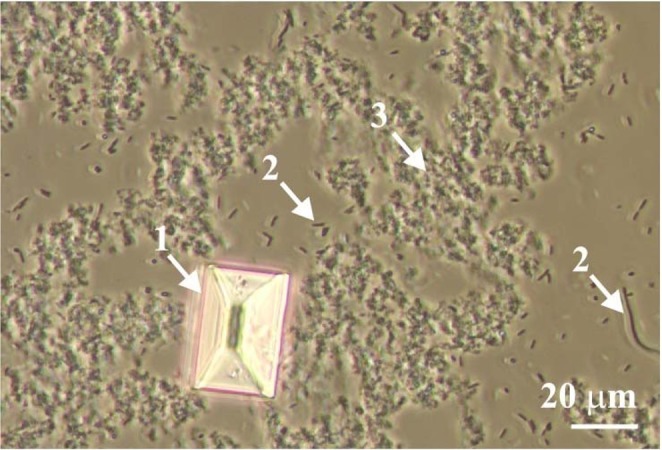
Struvite (arrow 1), bacteria *Proteus mirabilis* (arrow 2) and PCaAP (arrow 3) in the sample of artificial urine. The image was obtained under an optical light microscope with transmitted light. Figure reprinted from^12^.

Infectious urinary stones constitute a significant clinical problem that affects up to 20% of the population^1^, mainly in industrialized countries, and constitute between 10%^2^ and 30%^3^ of all human urinary stones. This type of urinary stones is formed as a result of urinary tract infections caused by urease-producing microorganisms^4^, mainly from the *Proteus* species^5^. Urease is a bacterial enzyme that accelerates the hydrolysis of urea, which is one of the components of the urine of a healthy person. As a result of this hydrolysis, ammonia and carbamic acid are first formed, which spontaneously decomposes to give carbonic acid and another molecule of ammonia. The resulting ammonia decomposes into ammonium and hydroxide ions. The appearance of hydroxide ions means an increase in the urine pH level. As a result of cascades of further reactions (described several times in the literature, for example in refs.^4,6–10^), appropriate ions and chemical complexes are formed, which lead to the crystallization of struvite (MgNH_4_PO_4_∙6H_2_O) and formation of poorly crystalline and amorphous precipitate (PCaAP). PCaAP is one of the main components of infectious urinary stones. Literature data indicate that the poorly crystalline phases include carbonate apatite Ca_10_(PO_4_)_6_CO_3_ (abbreviated as CA) and hydroxylapatite Ca_10_(PO_4_)_6_(OH)_2_ (abbreviated as HAP)^11,12^. CA and HAP may exist in non-stoichiometric forms, which means that groups CO_3_^2−^, OH^−^ and PO_4_^3−^ in CA and HAP may be substituted for other anions present in the urine. Non-stoichiometric forms include, for example, calcium sodium phosphate carbonate hydroxide, Ca_9_Na_0.5_(PO_4_)_4.5_(CO_3_)_1.5_(OH)_2_, and chlorapatite, Ca_10_(PO_4_)_6_Cl_2_ ^12^. The amorphous phases which are the part of infectious urinary stones include: amorphous calcium carbonate (ACC), amorphous calcium phosphate (ACP), and/or amorphous carbonated calcium phosphate (ACCP)^11–13^. All these poorly crystalline and amorphous phases are called briefly PCaAP. PCaAP (Fig. 1, arrow 3) does not show any visible features characteristic of crystalline solids and occurs in the form of clusters without a specific arrangement^11–13^. As stated above, during infection with urease-positive bacteria, a gradual increase in urine pH occurs. PCaAP begins to form when the urine pH reaches 6.8. For slightly higher urine pH (≥7.2), as a result of further chemical reactions induced by bacterial urease - magnesium ammonium phosphate hexahydrate (${{\rm{MgNH}}}_{4}{{\rm{PO}}}_{4}\,\cdot \,6{{\rm{H}}}_{2}{\rm{O}}$, struvite) crystallizes^10^. The size of struvite crystals along the crystallographic axis *b* reaches 100 μm. Struvite forms polyhedral crystals with specific crystallographic surfaces (Fig. 1, arrow 1). In living organisms (humans and animals), struvite crystals usually take on a coffin-like habit^14,15^.

Typically, the solid phases, PCaAP and struvite, formed in the urinary tract are small enough (several micrometers) to be excreted from the urinary tract along with the urine stream, without damaging the epithelial cells. Typically, the growth of a single crystal within the urinary tract does not lead to the formation of an infectious urinary stone. Such a stone is formed as a result of aggregation of a large amount of small struvite crystals and small PCaAP deposits. As indicated by literature^16^, urinary stones contain highly aggregated crystals. In addition, the bacteria can aggregate with themselves, as well as with struvite and PCaAP. So aggregated bacteria with struvite and PCaAP can be embedded in the stone structure^17^. Aggregation, as a much faster process than the growth of a single crystal, can very quickly lead to a large stone. Therefore, it is believed that aggregation is one of the main causes of urinary stones formation.

The aggregation process of struvite, PCaAP and microorganisms is studied in ref.^18^. However, in ref.^18^ PCaAP is called CA (carbonate apatite). During the research presented in ref.^18^, we did not study the exact composition of this precipitate, but we based on the literature (e.g. refs.^10,13,19^), in which it is assumed that the resulting precipitate is CA. After the research, the results of which we presented in ref.^12^ our knowledge about the phase composition of the resulting precipitate is greater and hence the name change for PCaAP.

Our results presented in ref.^18^ show that of the three components: struvite, PCaAP and microorganisms, PCaAP has the greatest ability to aggregate. In addition, our results presented in ref.^20^ demonstrate that in the presence of bacteria an increased aggregation process of PCaAP is observed. Experimental results indicate that among the macromolecules released from *Proteus mirabilis (P. mirabilis)* cells, lipopolysaccharide (LPS) is responsible for increased aggregation of PCaAP^20^. LPS is a main component of the outer membrane in Gram-negative bacteria and plays a role in their protection from adverse environmental conditions and it is an important virulence factor. Usually, LPS contains three parts in its structure: lipid A - specific carbohydrate lipid moiety that anchors LPS to the outer membrane; oligosaccharide core and the most external part of LPS having contact with the environment - antigen O (O- specific polysaccharide), connected to the core and composed of repeating oligosaccharide subunits^21^. There are many studies by other authors that suggest that, among large quantity of different bacterial factors, bacterial polysaccharides including LPS play an important role in the process of crystallization and infectious urinary stone formation^22–25^. For example, in refs.^21,25^, it is shown that, during infection caused by *Proteus*, LPS may accumulate Ca^2+^ and Mg^2+^ ions via electrostatic interactions and in this way accelerates the formation process of struvite and PCaAP. In ref.^25^ authors study metal binding by LPS extracted from strains of *Proteus mirabilis*, *Proteus vulgaris* and *Proteus penneri* and its influence on the struvite and PCaAP formation process. Results presented in ref.^25^ show that there are no significant differences between bacteria and its LPS interactions with Ca^2+^ and Mg^2+^ ions. From this result a conclusion is drawn that among the bacterial factors present on the cell surface LPS is mainly responsible for ions binding. Bacterial polysaccharides and LPS also influence the aggregation process of components precipitated in urine to form a stone^22^.

LPS due to the high biological activity and unusual physicochemical properties is subjected to numerous studies. As already mentioned, LPS is the main component of the outer bacterial membrane, so it is connected with the bacterial cell. Watson and co-workers^26^ demonstrated that, the amount of LPS present in the cell surface of *Escherichia coli* ranges from 28.9 fg to 49.4 fg per cell, depending on cell culture duration. During infection of the urinary tract caused by bacteria such as *P. mirabilis*, in addition to LPS occurring on live bacteria, free LPS is also detected in the urine. This is related to the disintegration of cells as a result of their death, but it also takes place in case of physiologically normal cells, for example, during their division and growth. It was found, for example, that the level of LPS in urine of patients with urinary tract infections ranges between 10–350 pg/ml when the number of bacteria is above 10^4^ CFU/ml^27^. It seems that this amount of LPS is sufficient to make this molecule play an active role in the formation of urinary stones as evidenced by the results of clinical trials. It has been shown that infectious urinary stones contain 36 times more LPS than those derived from metabolic disorders; e.g. *P. mirabilis* induced urinary stone contained 250 µg LPS per gram^28^. The literature review shows that researchers conduct LPS studies in different contexts, and the most commonly used is free LPS suspended in a suitable solvent over a wide concentration range from 2 μg/ml to 500 μg/ml^29–31^. In our previous work^20^ to assess the effect of *P. mirabilis* on PCaAP aggregation, we investigated the aggregation of PCaAP in the presence of bacterial macromolecules with and without LPS. In the case of bacterial macromolecules with LPS, the concentration of LPS was in the range 20–40 μg/ml. Such a concentration of LPS corresponds to the concentration of LPS in artificial urine after incubation with *P. mirabilis* for 24 h at 37 °C.

In the light of the above review of the literature and the results obtained by us^18,20^, the aim of the present study is to analyze the effect of LPS isolated from the *P. mirabilis* strain C11 on PCaAP aggregation and verify its ability to influence aggregation processes. LPS isolated from *P. mirabilis* was chosen for analysis because this species is isolated from human infectious urinary stones in 70% cases^32,33^. Based on the review of literature we investigated an influence of LPS of concentrations from 12 µg/ml to 440 µg/ml. In the present study the sizes and zeta potential values of PCaAP aggregates formed without bacteria but for different concentrations of LPS are compared with the sizes and zeta potential values of PCaAP formed in the presence of *P. mirabilis* as a function of pH.

We also present a specially designed experiment in which polystyrene latex beads with dimensions close to the size of bacteria are coated with LPS and thus mimic the presence of LPS-coated bacteria in the urine. The experiment designed in such a way should give the answer whether the influence on PCaAP aggregation has LPS freely suspended in artificial urine or connected with the bacterial cell. The goal of the present study is also understanding the role of LPS in PCaAP aggregation and finding relation between concentration of LPS and sizes of PCaAP aggregates.

## Materials and Methods

### Preparation of the artificial urine, bacterial suspension and LPS

Usually, the artificial urine used for various experiments is made from the following components^34^, with concentrations (g/l) in brackets: CaCl_2_∙2H_2_O, calcium chloride dihydrate (0.651), MgCl_2_∙6H_2_O, magnesium chloride hexahydrate (0.651), NaCl, sodium chloride (4.6), Na_2_SO_4_, sodium sulfate (2.3), KH_2_PO_4_, potassium dihydrogen phosphate (2.8), KCl, potassium chloride (1.6), NH_4_Cl, ammonium chloride (1.0), Na_3_C_6_H_5_O_7_, trisodium citrate (0.65), Na_2_C_2_O_4_, disodium oxalate (0.023), CO(NH_2_)_2_, urea (25.0), C_4_H_9_N_3_O_2_, creatine (1.1) and tryptic soy broth (10.0). Such a composition of artificial urine is widely accepted in literature^25,35^. In the present study we focus on the formation of PCaAP. Therefore, the composition of artificial urine was modified. The modification consists in this that the magnesium chloride hexahydrate (MgCl_2_·6H_2_O) was not added. This is related with the fact that the presence of magnesium chloride hexahydrate in artificial urine causes struvite precipitation, as described in Introduction, what is unwanted. Struvite would be able to disturb the spectrophotometric and zeta potential measurements. Such a composition of urine as well as the modification described has already been used by us in our previous studies and is originally described in ref.^36^.

The artificial urine of such a modified composition (Mg-free) was prepared by dissolving chemicals (Sigma Aldrich) of reagent-grade purity in distilled water. The further course of action with artificial urine is the same as procedure described in ref.^20^. This means, among other things, that the artificial urine was filtered using a membrane filter with pore size of 0.2 μm. It was stored for a maximum 48 h at 4 °C.

*P. mirabilis* strain C11 was obtained by courtesy of the Second Department of Urology, Medical University of Lodz, Poland and isolated from kidney stone of patient of this clinic. Before the crystal growth experiment, bacteria were maintained on a slant of tryptic soy agar overnight at 37 °C and then suspended in artificial urine to the concentration of 5∙10^5^ CFU per ml (the abbreviation CFU denotes colony forming unit). Precipitation of PCaAP occurs after addition of the suspension of bacteria and incubation at 37 °C. The precipitation process occurs at conditions emulating the natural conditions existing in human body during the infection by *P. mirabilis*.

LPS of *P. mirabilis* C11 was extracted from bacterial wall using hot phenol/water method. This method is described in detail in, for example, ref.^37^, but was developed by Westphal and Jann in 1965 and is originally described in ref.^38^. After extraction, LPS of *P. mirabilis* C11 was purified by treatment with cold aqueous 50% trichloroacetic acid, centrifugation and dialysis. The experiment with LPS is designed in two ways. In the first case, LPS is freely suspended in the solution of artificial urine. The tested concentrations of LPS are: 12 μg/ml, 50 μg/ml, 120 μg/ml, 200 μg/ml, 280 μg/ml, 360 μg/ml and 440 μg/ml. It should be noted that in the presence of *P. mirabilis* at a concentration of 5∙10^5^ CFU per ml, the concentration of LPS in the urine ranges between 20 and 40 μg/ml^20^. Hence, the investigated LPS concentrations of 12 μg/ml and 50 μg/ml are slightly below and above the LPS concentration limits in the presence of *P. mirabilis*. In the second case, LPS coats polystyrene latex beads of mean size 3 μm. Polystyrene latex beads were purchased form Sigma Aldrich. In order to cover polystyrene latex beads, they were incubated with LPS (in a ratio of 30 µl beads per 200 µg LPS) overnight at 4 °C in phosphate buffer pH 7.2 (PBS) with gentle stirring. Next, beads were washed three times in PBS to remove unbound LPS, and centrifuged each time (10,000 g for 10 minutes at 4 °C). After washing, the beads were suspended in the artificial urine to give the concentration of 5∙10^5^ beads/ml. In this way the polystyrene latex beads coated by LPS mimic the presence of bacteria in the artificial urine.

All experiments were performed using artificial urine of the same composition (modified, Mg-free). Control test is a sample of Mg-free artificial urine without *P. mirabilis*, LPS and polystyrene latex beads and without any other additives. In the case of the presence of *P. mirabilis* the precipitation process occurs as a result of urease activity; in all remaining cases the precipitation process of PCaAP occurs after gradual addition of aqueous ammonia solution (1.2 M). The aqueous ammonia solution is added in portions, not continuously. Such addition causes an increase in the pH and concentration of ammonium ions. In other words, the addition of aqueous ammonia solution mimics the activity of urease and thus emulates the real infection of the urinary tract caused by the bacteria. In all cases (control test, the experiments with the presence of *P. mirabilis*, the experiments with the presence of LPS or polystyrene latex beads) the experiments were conducted under thermostated conditions at 37 ± 0.5 °C. The temperature was kept constant by circulating water from a constant temperature water bath. This procedure has already been used by us in previous studies and is originally described in ref.^38^.

The pH of artificial urine was screened along the experiments using digital pH-meter (Elmetron CPC-401). The initial pH of artificial urine was adjusted to a value of 5.8. The value of pH increases with time because of urease activity in the case of presence of microorganisms or as a result of addition of aqueous ammonia solution in the case of absence of microorganisms. The value of pH equal to 9.5 is the highest value obtained during both kinds of experiments. In the case of the presence of *P. mirabilis* such a value is achieved after 8 h of incubation. The pH level is correlated with the bacterial viability, pH higher than 8 acts bactericide. Therefore, the number of live bacteria systematically decreases, and pH value does not exceed 9.5. In the case of the addition of aqueous ammonia solution the experiments are also performed up to pH equal to 9.5. The behavior of bacteria in urine and the increase in pH induced by the activity of bacterial urease is described in the literature, for example in ref.^20^. Imitating the behavior of bacteria in urine by adding aqueous ammonia solution has also been used in research and is described, for example, in ref.^20^.

### Spectrophotometric measurements and microscopic observations

In order to define the effect of LPS on PCaAP formation, the turbidity of artificial urine as an absorbance of light of defined wavelength was measured. The optimized wavelength in all cases was equal to 400 nm. This wavelength is optimal in respect of solid phases (PCaAP) produced in the artificial urine. Bacteria do not absorb this wavelength, therefore the absorbance does not increase with the increasing amount of bacteria. The measurements of turbidity were performed using spectrophotometer Spekol 11 (Carl Zeiss) and glass cuvettes with path length of 10 mm. Additionally, during all sets of experiments, the samples from different stages of precipitation process were observed with optical microscopy OptaTech MN 800. A drop of solution from the volume of artificial urine were taken and put in the middle of the micro-slide and then covering with a cover slip. The same procedure was used by us in our previous studies and is described originally in ref.^39^.

### Electrophoresis mobility and zeta potential measurements

Electrophoretic mobility measurement and zeta potential calculation of PCaAP were performed with Zeta-Meter 3.0+ system. The instrument consists of a measurement cell called electrophoresis cell. This cell is in a form of tube, 10 cm long and 4 mm in diameter. An electrical potential is applied to the electrodes at each end of the cell. The samples with investigated particles (PCaAP or/and LPS or/and coated polystyrene latex beads) were injected into the cell. These particles move in the electric field and are viewed and tracked with stereoscopic microscope with dark field illumination. This technique makes it possible to view micron-size particles without high-power magnification.

In the electrophoresis cell, the particles travel the reference distance. Based on the measured time taken by a particle to travel the reference distance, the velocity of the particle is estimated. The velocity of a particle in a unit of electric field is the electrophoretic mobility, μ, of a particle. On the basis of the electrophoretic mobility, μ, zeta potential, ζ, is calculated. Strictly speaking, in the case of our experiment, zeta potential, ζ, is determined using the Smoluchowski approximation^40^:

1 $${\rm{\zeta }}=\frac{{\rm{\mu }}\,{\rm{\eta }}}{{\rm{\varepsilon }}},$$

where ε and η is the dielectric constant and viscosity of the solution, respectively. In our case the electrophoretic mobility hence zeta potential of PCaAP is measured in the artificial urine. The viscosity of artificial urine used for calculation of zeta potential is equal to 0.83 cP^18^. The method of its determination is given in ref.^18^. The dielectric constant ε used for calculation is equal to 74.2^18^. This value of the dielectric constant corresponds to water at 37 °C.

PCaAP was obtained from Mg-free artificial urine as described in the previous subsection. The measurement of zeta potential followed directly after precipitation of this phase in the artificial urine. The artificial urine with PCaAP and/or with LPS and/or with coated polystyrene latex beads were sonicated in an ultrasonic bath (Sonic-3, Polsonic) for 3 minutes prior to measurement in order to ensure proper dispersion of the particles. This means that the zeta potential measurement was performed for the non-aggregated particles. All experiments were carried out under thermostated conditions at 37 ± 0.5 °C. All zeta potential measurements were taken at various pH from the range 7.5 to 9.5 in order to reflect the increasing pH during the real infection in urinary tract. Each experiment was repeated three times and the mean, standard deviation and relative standard deviation were calculated. Zeta potential of PCaAP in the presence of *P. mirabilis* was not measured.

### Method for measuring cross-sectional areas and calculating mean cross-sectional areas of PCaAP aggregates

To assess the effect of LPS on the PCaAP aggregation process, aggregate sizes were first analyzed based on microscopic images. The procedure for obtaining microscopic images was as follows. The drop of artificial urine with PCaAP was taken by pipette from the sample volume and placed on a micro-slide and covered with a coverslip. This means that the PCaAP aggregates located under the coverslip were observed rather as two-dimensional cross-sections, and not as three-dimensional forms. Therefore, the analysis of microscopic images makes it possible to measure the cross-sectional areas of aggregates. A detailed description of the method for measuring these cross-sectional areas of aggregates based on microscopic images is presented in ref.^20^.

Several series of measurements were carried out with *P. mirabilis*, with several concentrations of LPS and with polystyrene latex beads coated with LPS. The control test was always carried out for each measurement series. This means that there were a lot of control series. The runs of the control series, despite being carried out in the same way each time gave slightly different results. Cross-sectional areas (CSA) of PCaAP aggregates have always increased with increasing pH, however, not in the same way. In order to “average” the obtained results of the control series, the following actions were performed: the following CSA ratios were calculated for individual measurement series: ${{\rm{CSA}}}_{{\rm{pH}}=8.0}/{{\rm{CSA}}}_{{\rm{pH}}=7.5}$, ${{\rm{CSA}}}_{{\rm{pH}}=8.5}/{{\rm{CSA}}}_{{\rm{pH}}=8.0}$, ${{\rm{CSA}}}_{{\rm{pH}}=9.0}/{{\rm{CSA}}}_{{\rm{pH}}=8.5}$, ${{\rm{CSA}}}_{{\rm{pH}}=9.5}/{{\rm{CSA}}}_{{\rm{pH}}=9.0}$, then these ratios were averaged, rejecting extreme values. The mean cross-sectional areas of the PCaAP aggregates for individual pH values were calculated by multiplying the measured mean cross-sectional areas of the PCaAP aggregates by the calculated average CSA ratios defined above. In this way, a one “universal control test” was obtained, which is the same for all series of measurements. The control test shown in Fig. 3 is a “universal control test”.

From the point of view of determining the impact of LPS on the aggregation process, what is important is not the cross-sectional areas of the aggregates themselves, but their relation to cross-sectional areas in the control test, determining whether LPS at a given concentration enhances or inhibits the aggregation process at a given pH. To unambiguously present the results from the series with suspended LPS, beads coated with LPS and *P. mirabilis* in relation to “universal control test”, the following was done: for all series of measurements (with LPS, beads and bacteria) the following ratios were calculated: cross-sectional area at a given pH to cross-sectional area in a control test at the same pH (this is a control test for a given series, not a “universal control test”). The ratios obtained were averaged, not the cross-sectional areas themselves. Then, these averaged cross-sectional area ratios were scaled using “universal control test” to obtain the mean cross-sectional areas of the PCaAP aggregates for individual pH. Figure 3 shows mean cross-sectional areas of PCaAP aggregates calculated in this way.

The mean cross-sectional areas of PCaAP aggregates were determined in exactly the same way in our previous work^20^. In ref.^20^, the mean cross-sectional areas in the control test and in the presence of *P. mirabilis* are identical to those in the current work. The method used to calculate mean cross-sectional area of PCaAP aggregates helps in comparing the results presented in the current work with the results in ref.^20^ and in possible works published in the future. It should also be remembered that the systems considered in this work and in ref.^20^, especially in the presence of bacteria, are dynamic biological systems. In such systems, tendencies of changes are observed rather than absolute values only. Therefore, in this work we present the results of measurements of the cross-sectional areas of PCaAP aggregates in relation to the “universal control test”, and not absolute measurements with a standard deviation.

In addition, it should be noted that cross-sectional area measurements are only helpful in determining the effect of LPS on PCaAP aggregation and are only quantitative. Qualitative aggregation results can be obtained by measuring zeta potential. We also present such results in this work.

## Results

### Role of *P. mirabilis* and LPS in aggregation of PCaAP

**Figure 2:**
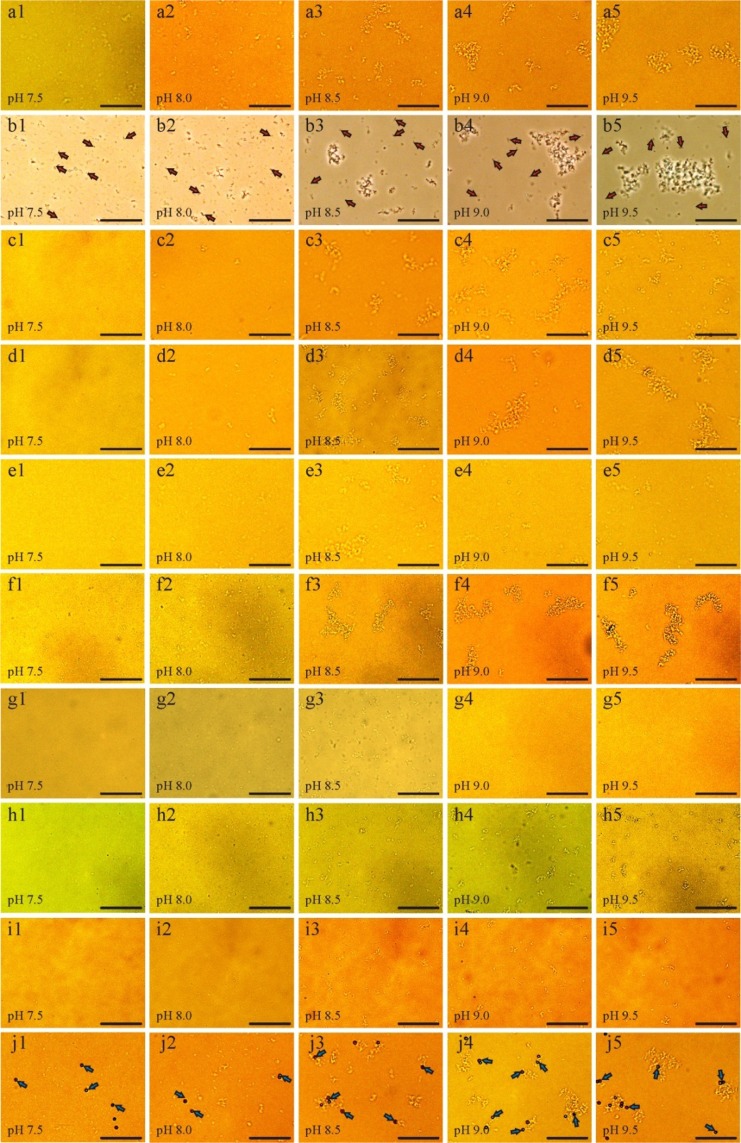
Aggregation process of PCaAP in the absence (a1–a5) and presence (b1 - b5) of *P. mirabilis* (arrows) and in the presence of LPS freely suspended in the artificial urine. LPS concentrations: 12 µg/ml (panels c1–c5); 50 µg/ml (panels d1–d5); 120 µg/ml (panels (e1–e5); 200 µg/ml (panels f1–f5), 280 µg/ml (panels g1–g5); 360 µg/ml (panels h1–h5) and 440 µg/ml (panels i1–i5). Panels j1–j5 present aggregation process of PCaAP in the presence of polystyrene latex beads (arrows) coated by LPS. The images’ quality is enhanced by the contrast and brightness to enable the identification of *P. mirabilis* and PCaAP. Scale bar: 40 μm.

The formation of PCaAP in artificial urine in the absence and presence of *P. mirabilis* depends strongly on pH value. The value of pH increases with time because of urease activity in the case of presence of microorganisms or as a result of addition of aqueous ammonia solution in the case of absence of microorganisms. Formation of PCaAP in the presence and absence of *P. mirabilis* has been the subject of our previous studies^18,20^. From the study presented in ref.^20^ it follows that the aggregation of PCaAP runs more efficiently in the presence of *P. mirabilis* than in the case of the absence of *P. mirabilis*. This means that microorganisms accelerate the aggregation of PCaAP. The results of our present experiments are presented in Fig. 2. Here we can see the processes of PCaAP formation depending on pH of artificial urine in the absence (a1-a5) and in the presence (b1-b5) of *P. mirabilis*. The results presented at Fig. 2, panels a1-a5 and b1-b5 confirm literature data that in the presence of *P. mirabilis* the aggregates of PCaAP are much larger than in the case of absence of *P. mirabilis*. In the presence of *P. mirabilis* PCaAP tends to form aggregates even for small pH, rather than to appear in small, free deposits (Fig. 2, panels b1-b5). The results presented in ref.^20^ suggest that for enhanced aggregation of PCaAP in the presence of *P. mirabilis*, LPS is responsible.

**Figure 3:**
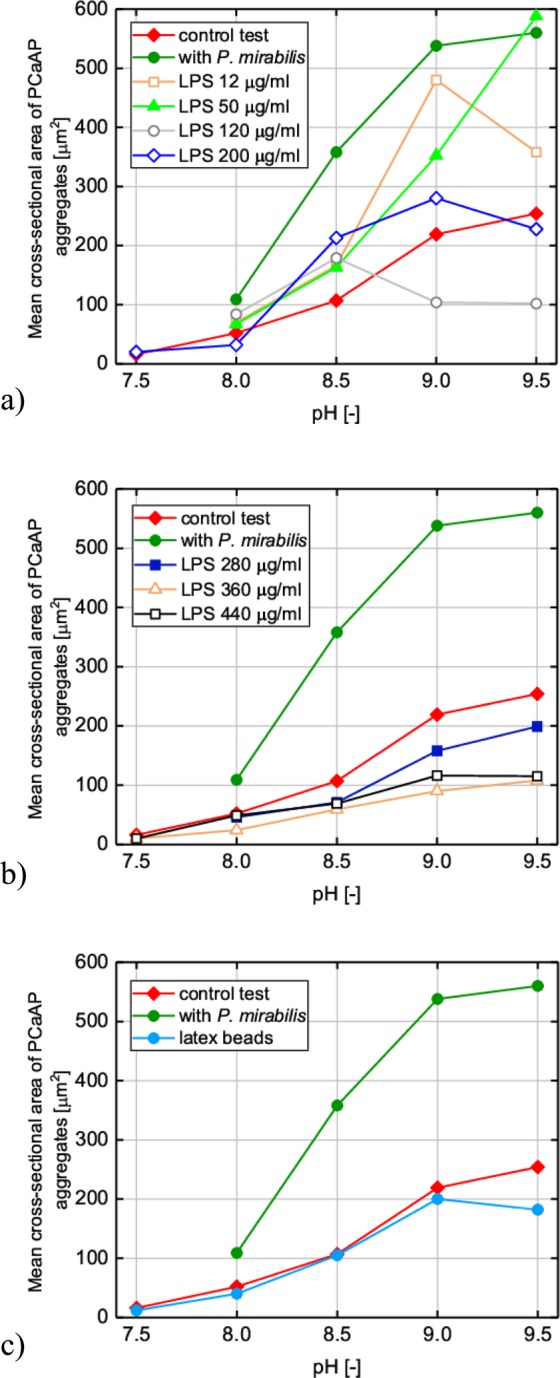
Mean cross-sectional area of PCaAP aggregates for control test (without *P. mirabilis* and without LPS) compared with mean cross-sectional area of PCaAP aggregates in the presence of (**a,b**) *P. mirabilis* and freely suspended LPS of different concentrations given in the insets and (**c**) LPS coated on polystyrene latex beads. In the case of *P. mirabilis* the value of mean cross-sectional area of PCaAP aggregates for pH = 7.5 is not given because for this pH, the agglomerations of bacteria are difficult to distinguish from PCaAP aggregates.

To verify the idea that LPS affects PCaAP aggregation, a series of experiments were carried out in the presence of LPS. In the case of LPS freely suspended in artificial urine the tested concentrations are equal to 12 μg/ml, 50 μg/ml, 120 μg/ml, 200 μg/ml, 280μg/ml, 360 μg/ml and 440 μg/ml. The results of our experiments are presented in Fig. 2 rows c, d, e, f, g, h, and i as microscopic images. Looking at these pictures it is difficult to evaluate the influence of LPS on the formation and aggregation of PCaAP. Based on these pictures we can only conclude that probably this influence depends on the concentration of LPS. To assess the effect of LPS on the PCaAP aggregation process, aggregate sizes were first analyzed based on microscopic images. In Fig. 3 PCaAP aggregate sizes are presented as mean cross-sectional areas of PCaAP aggregates. The method of calculating these mean cross-sectional areas is presented in Materials and Methods section.

The results shown in Fig. 3a confirm the microscopic observations described above, i.e. PCaAP aggregates are the largest in the presence of bacteria. Table 1 shows the detailed percentage changes in mean cross-sectional areas of PCaAP aggregates. From this Table it follows that in the presence of *P. mirabilis* the aggregates of PCaAP are larger than those in the control test (without LPS and without *P. mirabilis*) even by 234% for pH equal to 8.5.

**Table 1:** Percentage changes (%) in average cross-sectional area of PCaAP aggregates with respect to control test depending on pH in the presence of *P. mirabilis*, freely suspended LPS of different concentrations and LPS coated on polystyrene latex beads.

pH		7.5	8.0	8.5	9.0	9.5
*P. mirabilis*		*	+109	+234	+145	+120
freely suspended LPS of concentration in [µg/ml]	12	**	+34	+54	+119	+41
	50	**	+28	+52	+61	+131
	120	**	+60	+67	−52	−60
	200	+26	−39	+99	+28	−10
	280	**	−11	−33	−28	−22
	360	−34	−54	−45	−59	−57
	440	−38	−6	−36	−47	−55
polystyrene latex beads coated by LPS		−24	−24	−2	−9	−28

*In the case of *P. mirabilis* the percentage change for pH = 7.5 is not given, because for this pH, the agglomerations of bacteria are difficult to distinguish from PCaAP aggregates.

**For these concentrations of LPS and pH equal to 7.5 PCaAP is formed in very small amount making impossible the measurement of cross-sectional area of PCaAP aggregates.

The mean cross-sectional areas of PCaAP aggregates in the presence of LPS freely suspended in the artificial urine are presented in Fig. 3a,b. Figure 3a is related to the small and intermediate concentrations of LPS, while Fig. 3b is related with high concentration of LPS. From Fig. 3a it is seen that for the smallest tested concentrations of LPS equal to 12μg/ml and 50 μg/ml the mean cross-sectional areas of PCaAP aggregates are larger compared to control test. As follows from Table 1 the aggregates of PCaAP in the presence of LPS of concentration equal to 12 μg/ml are larger than those in the control test even by 119% for pH = 9.0. For concentrations of LPS equal to 12 μg/ml and 50 μg/ml the cross-sectional areas of PCaAP aggregates are comparable with those in the case of presence of *P. mirabilis* for high pH equal to 9.0 and 9.5 (Fig. 3a). For pH equal to 8 and 8.5 the aggregates of PCaAP are smaller than those in the presence of *P. mirabilis* by 45% and 47% for LPS concentration equal to 12μg/ml and 50 μg/ml, respectively (Fig. 3a). However, for pH equal to 9 and 9.5 the aggregates of PCaAP are smaller than those in the presence of *P. mirabilis* by only 23% and 16% for LPS concentration equal to 12 μg/ml and 50 μg/ml, respectively. For pH equal to 9.5, the aggregates of PCaAP are even larger than those in the presence of *P. mirabilis* by 5% for LPS concentration equal to 50 μg/ml (Fig. 3a). These results suggest that LPS of concentration equal to 12 μg/ml and 50 μg/ml enhance the aggregation of PCaAP when compared with control test.

For higher concentrations of LPS equal to 120 μg/ml and 200 μg/ml the mean cross-sectional areas of PCaAP aggregates (Fig. 3a) are significantly smaller than those in the presence of *P. mirabilis*. In this case we may say that the cross-sectional areas of PCaAP aggregates are closer to those in the case of control test than those in the presence of *P. mirabilis*. For the highest tested concentrations of LPS equal to 280 μg/ml, 360 μg/ml and 440 μg/ml the aggregates of PCaAP are the smallest compared with other tested concentrations of LPS (Fig. 3b). In this case the cross-sectional areas of PCaAP aggregates are much smaller than those in the control test for the whole range of pH. This means that LPS with the highest concentration tested inhibits PCaAP aggregation as compared to the control test.

The presented results show that LPS demonstrates three-phase activity depending on its concentration. For small concentrations (12 μg/ml and 50 μg/ml), LPS enhances the aggregation of PCaAP when compared to control test. For these concentrations of LPS the cross-sectional areas of PCaAP aggregates are comparable with those in the case of presence of *P. mirabilis* for high pH equal to 9.5. For intermediate concentrations of LPS equal to 120 μg/ml and 200 μg/ml the aggregates of PCaAP are of comparable cross-sectional areas with those in the control test and, at the same time, much smaller than those in the presence of *P. mirabilis*. For highest tested concentrations equal to 280 μg/ml, 360 μg/ml and 440 μg/ml, LPS inhibits the formation of PCaAP aggregates. For these concentrations of LPS, the cross-sectional areas of aggregates of PCaAP are smaller than those in the control test and much smaller than those in the presence of *P. mirabilis*. However, it should be remembered that the described results concern the situation when LPS is freely suspended in the artificial urine. In reality, as described in the Introduction, LPS is detected in the urine, but it also occurs on the surface of bacterial cells. Considering this fact that LPS is not only detected in the urine, but also occurs on the surface of bacterial cells, a second type of experiment was thinked of.

This kind of experiment, as described in Materials and Methods Section, consists on this that LPS coats polystyrene latex beads. In this way, the polystyrene latex beads coated by LPS mimic the presence of bacteria in the artificial urine. Figure 3c presents the comparison of the cross-sectional areas of PCaAP aggregates in the case of the presence of *P. mirabilis* with control test and the case in which polystyrene latex beads are coated by LPS. On the basis of Fig. 3c we may conclude that in the case of LPS coated on the polystyrene latex beads the mean cross-sectional area of PCaAP aggregates is comparable with that in the case of control test (without *P. mirabilis* and without LPS); see also Table 1. The measurements of mean cross-sectional area of PCaAP aggregates are in agreement with microscopic observations presented in Fig. 2, panels j1-j5. Here, we can see that the course of aggregation of PCaAP in this case is very similar to that in the case of control test; Fig. 2, panels a1-a5.

From these results it follows that the influence of LPS on the average cross-sectional areas of PCaAP aggregates depends on this whether they are freely suspended in the artificial urine or coated on the polystyrene latex beads. When LPS is freely suspended in the artificial urine it is in the form of long chains consisting of a lipid and a polysaccharide. In such a form, for higher concentrations, LPS may coat easily small PCaAP deposits and not allow forming of large aggregates. The greater the concentration of LPS the more intensely LPS inhibits the aggregation of PCaAP. LPS which is coated on the polystyrene latex beads has no such properties because LPS adheres to the surface of these beads and does not pass to the artificial urine.

### PCaAP aggregation characterized by zeta potential

**Figure 4:**
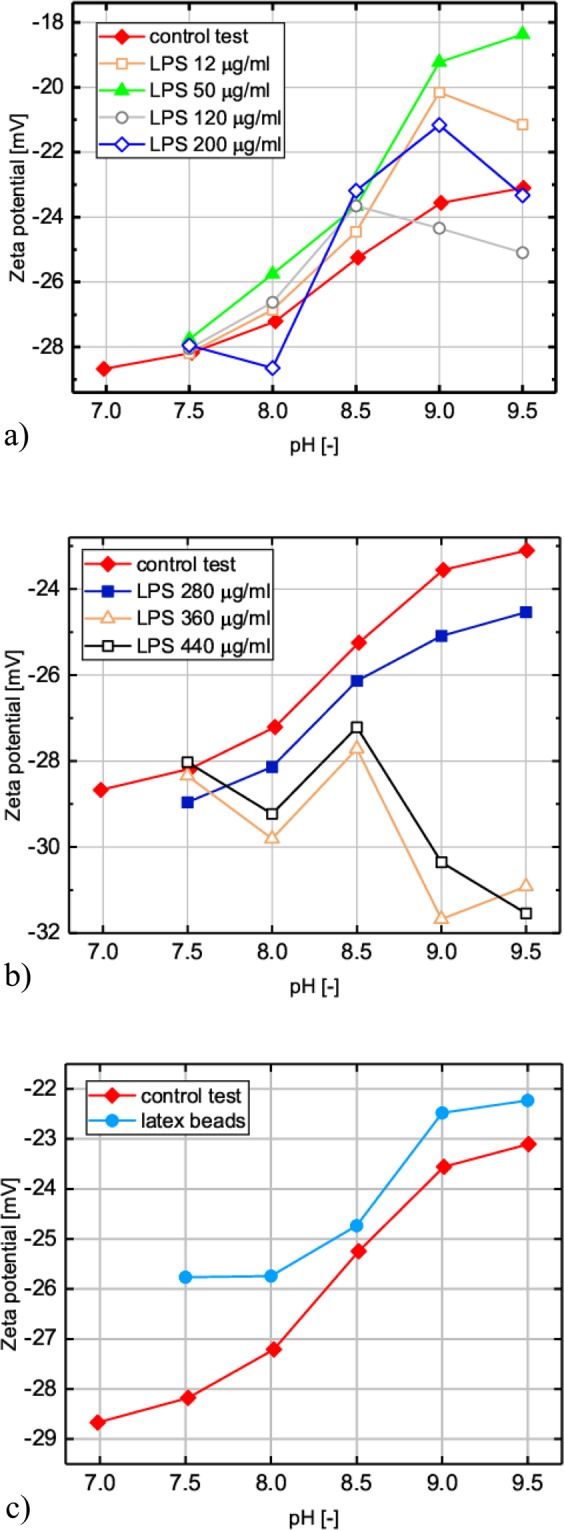
Zeta potential of PCaAP in the presence of (**a,b**) freely suspended LPS of different concentrations given in the insets and (**c**) polystyrene latex beads coated by LPS.

Figure 4 presents zeta potential of PCaAP depending on pH in the case of control test, in the presence of freely suspended LPS (Fig. 4a,b) in the Mg-free artificial urine and in the case when LPS is coated on polystyrene latex beads (Fig. 4c). Table 2 gives the zeta potential, ζ, standard deviation, SD, and relative standard deviation, RSD, for each measuring point shown in Fig. 4. The RSD reaches values from a few to several percent. For example, in the case of a control test for pH = 7.5 RSD is equal to 12.9%. It seems that this is due to the fact that for this pH the amount of PCaAP formed is very small and measuring the zeta potential is very difficult in this case. In general, it can be assumed that both SD and RSD values are within reasonable limits and are standard, taking into account that the system under consideration is a complex and dynamic biological system. In the case of some concentrations of LPS for pH equal to 7.5 zeta potential of PCaAP is not given (Fig. 4 and Table 2). This is because, in these cases, PCaAP is formed in very small amount making impossible the measurement of zeta potential. On the basis of Fig. 4 one can see that for control test zeta potential of PCaAP increases slightly with pH. For the lowest value of pH equal to 7 zeta potential takes the lowest value equal to ‒28.7 mV. With increase in pH, zeta potential also increases and for the highest value of pH equal to 9.5 it takes value of ‒23.1 mV.

**Table 2:** Zeta potential, ζ, standard deviation, SD, and relative standard deviation, RSD, of PCaAP for control test and in the presence of LPS of different concentrations and in the presence of LPS coated on polystyrene latex beads.

			pH					
			7.0	7.5	8.0	8.5	9.0	9.5
Control Test		ζ [mV]	−28.7	−28.2	−27.2	−25.2	−23.6	−23.1
		SD [mV]	3.7	2.0	2.3	1.2	1.8	1.5
		RSD [%]	12.9	7.0	8.5	4.8	7.6	6.5
freely suspended LPS of concentration in [µg/ml]	12	ζ [mV]	*	−28.2	−26.9	−24.5	−20.2	−21.2
		SD [mV]	*	1.8	1.5	1.3	1.0	0.6
		RSD [%]	*	6.4	5.6	5.3	5.0	2.8
	50	ζ [mV]	*	−27.8	−25.8	−23.7	−19.2	−18.4
		SD [mV]	*	1.4	1.2	2.3	2.8	1.0
		RSD [%]	*	5.0	4.7	9.7	14.4	5.6
	120	ζ [mV]	*	−28.1	−26.6	−23.7	−24.3	−25.1
		SD [mV]	*	2.0	2.0	1.1	1.7	1.4
		RSD [%]	*	7.1	7.5	4.7	6.9	5.5
	200	ζ [mV]	*	−27.9	−28.9	−23.2	−21.2	−23.3
		SD [mV]	*	3.3	2.8	2.8	2.7	2.6
		RSD [%]	*	11.8	9.9	11.9	12.7	11.1
	280	ζ [mV]	*	−29.0	−28.1	−26.1	−25.1	−24.5
		SD [mV]	*	1.1	1.1	1.6	1.3	1.6
		RSD [%]	*	3.6	3.8	6.0	5.1	6.5
	360	ζ [mV]	*	−28.4	−29.8	−27.7	−31.7	−30.9
		SD [mV]	*	2.7	1.1	3.0	2.3	1.2
		RSD [%]	*	9.5	3.6	10.8	7.3	3.9
	440	ζ [mV]	*	−28.0	−29.2	−27.2	−30.4	−31.5
		SD [mV]	*	1.3	1.3	1.3	1.2	1.3
		RSD [%]	*	4.8	4.4	4.9	4.0	4.0
PCaAP with latex beads		ζ [mV]	*	−25.8	−25.7	−24.7	−22.5	−22.2
		SD [mV]	*	2.2	1.7	1.5	1.3	1.3
		RSD [%]	*	8.5	6.5	5.9	6.0	6.0

*In these systems PCaAP is formed in very small amount making zeta potential measurement impossible.

Zeta potential of PCaAP in the presence of freely suspended LPS of low concentrations equal to 12 μg/ml and 50 μg/ml takes higher values (less negative) than those for PCaAP in control test; Fig. 4a. The results demonstrate that zeta potential for PCaAP in the case of LPS of concentration equal to 50 μg/ml takes the highest value equal to ‒18.4 mV for pH = 9.5. In comparison with control test for the same pH = 9.5 this zeta potential value is higher (less negative) by 36%. In the case of intermediate concentrations of LPS equal to 120 μg/ml and 200 μg/ml zeta potential of PCaAP takes smaller values (more negative) compared with the concentrations of LPS equal to 12 μg/ml and 50 μg/ml; Fig. 4a. For the highest concentrations of LPS equal to 280μg/ml, 360 μg/ml and 440 μg/ml zeta potential of PCaAP takes smaller vales (more negative) compared to control test; Fig. 4b. In particular, for concentration of LPS equal to 440 μg/ml for pH = 7.5 zeta potential of PCaAP is comparable with those for the control test; Fig. 4b. However, with increase in pH zeta potential of PCaAP for this concentration of LPS becomes more and more negative taking the smallest value equal to ‒31.5 mV for pH equal to 9.5; Fig. 4b.

The situation is different when LPS coats polystyrene latex beads; Fig. 4c. Zeta potential of PCaAP in the presence of so prepared beads displays values very close to those in the case of control test. With increase in pH zeta potential of PCaAP slightly increases (becomes less negative).

Zeta potential is a crucial indicator of the stability of the particle suspension. As a rule, suspensions of particles with ζ satisfying the ‒30 mV > ζ  > 30 mV condition are considered stable^41,42^.This means that suspensions of particles having ζ satisfying this condition are stable, whereas suspensions of particles with ζ in the 〈−30 mV, +30 mV〉 range tend to aggregate. Taking into account this criterion we may say that the suspension of PCaAP is the most stable for low pH up to 7.5 for which zeta potential is close to ‒30 mV. With increase in pH, zeta potential of the suspension of PCaAP for control test (Fig. 4a), for the concentration of LPS equal to 12 μg/ml, 50 μg/ml (Fig. 4a) and for the suspension of PCaAP in the presence of polystyrene latex beads (Fig. 4c), becomes less negative. Therefore, the suspension of PCaAP becomes more unstable with respect to aggregate. This means that, for these cases, PCaAP aggregates much more efficiently. In the case of higher concentrations of LPS equal to 360 μg/ml and 440 μg/ml (Fig. 4b) zeta potential of the suspension of PCaAP decreases (becomes more negative) with increasing in pH. This means that for this case PCaAP does not tend to aggregate.

In conclusion, the results of zeta potential measurements indicate that the effect of LPS on PCaAP aggregation depends on the LPS concentrations. The course of variation of the zeta potential in the presence of LPS with the lowest tested concentrations equal to 12 μg/ml and 50 μg/ml is comparable with that in the presence of *P. mirabilis*. This means that LPS at concentrations 12 μg/ml and 50 μg/ml enhances PCaAP aggregation. LPS with higher concentrations (360 μg/ml and 440 μg/ml) does not show aggregation enhancing properties. On the contrary, LPS with higher concentrations inhibits PCaAP aggregation. These results are consistent with our microscopic observations (Fig. 2) and measurements of cross-sectional area of aggregates (Fig. 3).

Let us focus for a moment on the composition of artificial urine, which we use for our research. As mentioned in Materials and Methods, we use modified urine without MgCl_2_∙6H_2_O. Therefore, one could ask whether the lack of MgCl_2_∙6H_2_O in artificial urine has an impact on the zeta potential and, consequently, on the aggregation of PCaAP. Lack of MgCl_2_∙6H_2_O can affect the distribution of ions in artificial urine and its conductivity, and conductivity affects the zeta potential^43^. The conductivity of artificial urine for normal and modified composition was studied in ref.^18^. These studies show that the conductivity of artificial urine without MgCl_2_∙6H_2_O is slightly lower compared to urine of normal composition. To be precise, the conductivity of urine without MgCl_2_∙6H_2_O in the pH range of 7 to 9.5 is lower than the conductivity of urine with normal composition by an average of 0.13 mS/cm, which is 0.68% of the average value of urine conductivity with a normal composition in this pH range. Such small changes in conductivity should not affect the zeta potential and consequently PCaAP aggregation. Therefore, our results of zeta potential measurements should be considered reliable and correct also in the case of urine with normal composition.

### Influence of LPS on the nucleation and growth of PCaAP

**Figure 5:**
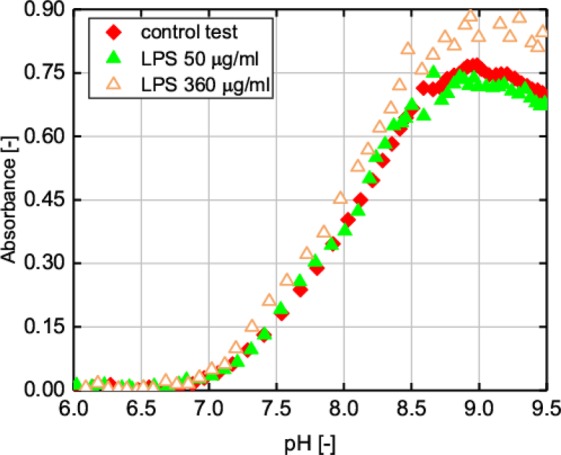
Dependence of absorbance of artificial urine versus pH for concentration of LPS equal to **(a**) 50 µg/ml and (**b**) 360 µg/ml. The graphs concern the formation of PCaAP. The gradual increase in absorbance is related with the precipitation of PCaAP. Symbols ,  and  correspond to the aggregation process shown in Fig. 2 in rows a, d and h, respectively.

The results presented above show that the freely suspended LPS affects PCaAP aggregation. One may wonder whether the freely suspended LPS affects the precipitation of PCaAP, i.e. the amount of PCaAP formed and the pH at which PCaAP begins to form. To analyze this problem the spectrophotometric measurements were performed. In particular, the turbidity of artificial urine with and without LPS as the absorbance of light of the defined wavelength (400 nm) was measured. Figure 5 presents our spectrophotometric results i.e. the absorbance as a function of pH for a control test (without *P. mirabilis* and without LPS) and for two exemplary LPS concentrations (50 μg/ml and 360 μg/ml). The measuring points shown in Fig. 5 come from one example series. This series is representative of all measured series. The nature of the absorbance changes depending on the pH is the same for all series. The gradual increase in absorbance is due to the formation of solid phase in the artificial urine, strictly speaking the PCaAP formation. On the basis of Fig. 5 one may notice that in the case of low LPS concentration equal to 50 μg/ml the increase in absorbance takes place for the same pH as in the case of control test. Additionally, the maximum value of absorbance is the same as for control test. This means that the precipitation of PCaAP in the presence of LPS of concentration equal to 50 μg/ml proceeds in the same manner as for the control test. In other words, LPS freely suspended in the artificial urine of concentration equal to 50 μg/ml does not influence on the precipitation process of PCaAP. The situation is a little different in the case of LPS of concentration equal to 360 μg/ml. In this case, based on Fig. 5, one may notice that the gradual increase in absorbance takes place for the same pH as in the case of control test. This means that the presence of LPS in this concentration does not influence on the nucleation of PCaAP. However, beginning form pH = 7.2 the absorbance is slightly higher (Fig. 5) than in the case of control test. Additionally, the maximum value of absorbance is slightly higher compared to the control test. This means that in the presence of LPS of concentration equal to 360 μg/ml the amount of formed PCaAP is a little greater (by 16%) than in the case of control test. The similar tendency is observed also for the concentration equal to 440 μg/ml (data not shown in Fig. 5). The higher the concentration of LPS, the more pronounced the described tendency is. This observation can be explained in the same way as in ref.^20^. In ref.^20^ the problem of PCaAP aggregation in the presence of bacterial macromolecules with and without LPS is undertaken. In ref.^20^, we showed an increase in the maximum absorbance value in the presence of bacterial macromolecules with LPS.

In ref.^20^ we suggest that LPS can serve as a matrix giving favorable sites for PCaAP nucleation and formation. This suggestion results from the similarity to the case described in the literature^44–46^ and concerning the study of polysaccharides as a matrix for precipitation of solid phases. The authors of articles^44–46^ indicate that polysaccharides can play the role of nucleation sites and can mediate the development of these solid phases. This is due to the fact that polysaccharides can lower the energy barrier for nucleation and thus accelerate nucleation^44,45^. Polysaccharides are part of LPS, therefore we suggest that in the case observed in this study, LPS may act similarly to polysaccharides and mediate the development of PCaAP and increase the maximum amount of PCaAP. The interaction between LPS and precipitated PCaAP can take place by means of vander Waals forces or hydrogen bonds. This effect has been described for polysaccharides^45,47,48^. We suggest that in the present study, the increase in maximum absorbance at elevated LPS concentrations can be explained in the same way. In the same manner we explain the increase in the amount of PCaAP formed in the presence of bacterial macromolecules with LPS in ref.^20^.

## Conclusions

From among many bacterial factors which may have an influence on the aggregation of PCaAP, in the present study, an influence of LPS is investigated. The obtained results show that LPS has an impact on PCaAP aggregation. This effect depends on the concentration of LPS and on whether LPS is freely suspended in artificial urine or coats latex beads that simulate the presence of bacteria. Freely suspended LPS of low concentrations (12 μg/ml, 50 μg/ml), enhances PCaAP aggregation. For such concentrations of LPS, the cross-sectional areas of PCaAP aggregates are comparable to those in the presence of bacteria. The LPS concentrations of these orders are those found during urinary tract infections with *P. mirabilis*. This result means that in the case of *P. mirabilis* infection LPS is responsible for PCaAP aggregation. The presented investigations provide evidence that aggregation processes of PCaAP are subject to biological regulations.

In the case of high concentrations of freely suspended LPS (360 μg/ml, 440 μg/ml), the formation of PCaAP aggregates is inhibited. It should be noted, however, that such high concentrations do not occur during urinary tract infections. The possible mechanism of inhibition of aggregation by high-concentration LPS may be as follows: LPS freely suspended in the artificial urine is in the form of long chains consisting of a lipid and a polysaccharide and in such a form, LPS of higher concentrations, coats easily small deposits of PCaAP and does not allow forming of large aggregates. The higher the concentration of LPS the more intensely LPS inhibits the aggregation of PCaAP.

LPS which is coated on the polystyrene latex beads has no effect on PCaAP aggregation. In this case LPS adheres to the surface of these beads and does not pass to the solution of artificial urine. In other words, only LPS freely suspended in artificial urine affects the PCaAP aggregation.

In summary, it was found that the LPS is involved in the aggregation of PCaAP during infectious urinary stones formation and this effect depends on its concentration in urine. Aggregation of solid phases is a very important step in the formation of urinary stones because resulting agglomerates can easily stop in the urinary tract and continue to grow to form stone. To date, there is little information on the aggregation mechanism and factors responsible for this process in the context of formation of infectious urinary stones. Knowledge on this subject may have a practical application in the treatment or prevention of the urolithiasis by seeking aggregation inhibitors. This approach has already found its application. For example, the study of aggregation mechanisms contributed to finding a substance that inhibits the aggregation of calcium oxalate crystals^49^.

## Data Availability

The datasets analysed during the current study are currently not publicly available, they held by the authors.

## References

[1] Benramdane L, Bouatia M, Idrissi MOB, Draoui M (2008). Infrared analysis of urinary stones, using a single reflection accessory and a KBr pellet transmission. Spectrosc. Lett..

[2] Aggarwal, K. P., Narula, S., Kakkar, M. & Tandon, C. Nephrolithiasis: Molecular Mechanism of Renal Stone Formation and the Critical Role Played by Modulators. *Biomed Res. Int*., Article ID 292953, 21 pages (2013). 10.1155/2013/292953 PMC3787572 24151593

[3] Chauhan CK, Joshi MJ (2013). *In vitro* crystallization, characterization and growth-inhibition study of urinary type struvite crystals. J. Cryst. Growth.

[4] Bichler KH (2002). Urinary infection stones. Int. J. Antimicrob. Ag..

[5] Prywer, J. & Torzewska, A. Effect of Curcumin Against *Proteus mirabilis* During Crystallization of Struvite from Artificial Urine. *Evid. Based Complement. Alternat. Med*. Article ID 862794, 7 pages (2012). 10.1155/2012/862794 PMC3144717 21808656

[6] Prywer J, Olszynski M (2013). Influence of disodium EDTA on nucleation and growth of struvite and carbonate apatite. J. Cryst. Growth.

[7] Amtul Z (2002). Chemistry and mechanism of urease inhibition. Curr. Med. Chem..

[8] Ch. Bouropoulos N, Koutsoukos PG (2000). Spontaneous precipitation of struvite from aqueous solutions. J. Cryst. Growth.

[9] Prywer J, Mielniczek-Brzóska E (2016). Chemical equilibria of complexes in urine. A contribution to the physicochemistry of infectious urinary stone formation. Fluid Phase Equilibr..

[10] McLean RJC, Nickel JC, Cheng KJ, Costerton JW (1988). The ecology and pathogenicity of urease-producing bacteria in the urinary tract. Crit. Rev. Microbiol..

[11] Grases F, Söhnel O, Vilacampa AI, March JG (1996). Phosphates precipitating from artificial urine and fine structure of phosphate renal calculi. Clin. Chim..

[12] Prywer J, Kozanecki M, Mielniczek-Brzóska E, Torzewska A (2018). Solid Phases Precipitating in Artificial Urine in the Absence and Presence of Bacteria *Proteus mirabilis*−A Contribution to the Understanding of Infectious Urinary Stone Formation. Crystals.

[13] Carpentier X (2009). Relationships Between Carbonation Rate of Carbapatite and Morphologic Characteristics of Calcium Phosphate Stones and Etiology. Urology.

[14] Dominick MA (2006). Urothelial Carcinogenesis in the Urinary Bladder of Male Rats Treated with Muraglitazar, a PPARα/γ Agonist: Evidence for Urolithiasis as the Inciting Event in the Mode of Action. Toxicol. Pathol..

[15] Abbona F, Boistelle R (1979). Growth Morphology and Crystal Habit of Struvite Crystals (MgNH_4_PO_4_∙6H_2_O). J. Cryst. Growth.

[16] Hess B, Nakagawa Y, Coe FL (1989). Inhibition of calcium oxalate monohydrate crystal aggregation by urine proteins. Am. J. Physiol..

[17] Li X (2002). Visualization of *Proteus mirabilis* within the Matrix of Urease-Induced Bladder Stones during Experimental Urinary Tract Infection. Infect Immun..

[18] Prywer J, Sadowski RR, Torzewska A (2015). Aggregation of Struvite, Carbonate Apatite, and *Proteus mirabilis* as a Key Factor of Infectious Urinary Stone Formation. Cryst. Growth Des..

[19] Gleeson MJ, Griffith DP (1993). Struvite calculi. Br. J. Urol..

[20] Prywer J, Torzewska A (2019). Impact of bacteria on aggregation of crystalline and amorphous components of infectious urinary stones. J. Cryst. Growth.

[21] Rozalski A, Sidorczyk Z, Kotelko K (1997). „Potential virulence factors of *Proteus* Bacilli. Microbiol. Mol. Biol. R..

[22] McLean RJC, Nickel JC, Beveridge TJ, Costerton JW (1989). Observations of the ultrastructure of infected kidney stones. J. Med. Microbiol..

[23] Clapham L, McLean RLC, Nickel JC, Downey J, Costerton JW (1990). The influence of bacteria on struvite crystal habit and its importance in urinary stone formation. J. Cryst. Growth.

[24] Dumanski AJ, Hedelin H, Edin-Liljegren A, Beauchemin D, McLean RJC (1994). Unique ability of the *Proteus mirabilis* capsule to enhance mineral growth in infectious urinary calculi. Infect. Immun..

[25] Torzewska A, Stączek P, Różalski A (2003). Crystallization of urine mineral components may depend on the chemical nature of *Proteus* endotoxin polysaccharides. J. Med. Microbiol..

[26] Watson SW, Novitsky TJ, Quinby HL, Valois FW (1977). Determination of bacterial number and biomass in the marine environment. Appl. Environ. Microbiol..

[27] Matsumoto T (1991). Significance of urinary endotoxin concentration in patients with urinary tract infection. Urol. Res..

[28] Gomez-Nunez, J. G. *et al*. Infected urinary stones, endotoxins and urospesis, clinical management of complicated urinary tract infection”, Nikibakhsh, A. (Ed.) ISBN:978-953-307-393-4, InTech, 183–198 (2011).

[29] Choi J, Klinkspoor JH, Yoshida T, Lee SP (1999). Lipopolysaccharide From *Escherichia coli* Stimulates Mucin Secretion by Cultured Dog Gallbladder Epithelial Cells. Hepatology.

[30] Romero C, Benito E, Bosch MA (1995). Effect of *Escherichia coli* lipopolysaccharide on surfactant secretion in primary cultures of rat type II pneumocytes. Biochim. Biophys. Acta.

[31] Liu MJ (2014). The protective effect of caffeic acid against inflammation injury of primary bovine mammary epithelial cells induced by lipopolysaccharide. J. Dairy Sci..

[32] Lerner SP, Gleeson MJ, Griffith DP (1989). Infection stones. J. Urol..

[33] Kramer G, Klinger HC, Steiner GE (2000). Role of bacteria in the development of kidney stones. Curr. Opin Urol..

[34] Griffith DP, Musher DM, Itin C (1976). Urease. The primary cause of infection-induced urinary stones. Invest. Urol..

[35] Prywer J, Torzewska A, Płociński T (2012). Unique Surface and Internal Structure of Struvite Crystals Formed by *Proteus mirabilis*. Urol. Res..

[36] Prywer J, Olszynski M, Mielniczek-Brzóska E (2015). Inhibition of precipitation of carbonate apatite by trisodium citrate analysed in base of the formation of chemical complexes in growth solution. J. Solid State Chemistry.

[37] Wang, X., Zhang, C., Shi, F. & Hu, X. Purification and Characterization of Lipopolysaccharides. In: Wang, X. & Quinn, P. (eds) Endotoxins: Structure, Function and Recognition. Subcellular Biochemistry, vol 53, pp 27–51, Springer, Dordrecht (2010). 10.1007/978-90-481-9078-2_2 20593261

[38] Westphal O, Jann K (1965). Bacterial lipopolysaccharide. Extraction with phenol-water and further applications of the procedure. Methods Carbohydr. Chem..

[39] Prywer J, Olszynski M (2013). Influence of disodium EDTA on the nucleation and growth of struvite and carbonate apatite. J. Cryst. Growth.

[40] Wilson WW, Wade MM, Holman SC, Champlin FR (2001). Status of methods for assessing bacterial cell surface charge properties based on zeta potential measurements. J. Microbiol. Methods.

[41] Mekhamer WK (2010). The colloidal stability of raw bentonite deformed mechanically by ultrasound. Saudi Chem. Soc..

[42] Uskokovic V (2012). Dynamic Light Scattering Based Microelectrophoresis: Main Prospects and Limitations. J. Dispersion Sci. Technol..

[43] Moran JL, Posner JD (2014). Role of solution conductivity in reaction induced charge auto-electrophoresis. Phys. Fluids.

[44] Ehrlich H (2010). Chitin and collagen as universal and alternative templates in biomineralization. Int. Geol. Rev..

[45] Yang L (2003). Interfacial molecular recognition between polysaccharides and calcium carbonate during crystallization. J. Inorg. Biochem..

[46] Arias JL, Fernandez MS (2008). Polysaccharides and proteoglycans in calcium carbonate- based biomineralization. Chem. Rev..

[47] Liu XY, Lim SW (2003). Templating and supersaturation-driven anti-templating: Principles of biomineral architecture. J. Am. Chem. Soc..

[48] Kontrec J, Kralj D, Brecevic L, Falini G (2008). Influence of some polysaccharides on the production of calcium carbonate filler particles. J. Cryst. Growth.

[49] Yan O (2015). Inhibition of Urinary Macromolecule Heparin on Aggregation of Nano-COM and Nano-COD Crystals. Molecules.

