# Publisher Correction: Aggregation of poorly crystalline and amorphous components of infectious urinary stones is mediated by bacterial lipopolysaccharide

**DOI:** 10.1038/s41598-020-58429-1

**Published:** 2020-01-27

**Authors:** Jolanta Prywer, Agnieszka Torzewska

**Affiliations:** 1 Institute of Physics, Lodz University of Technology, ul. Wólczańska 219, 90-924 Łódź, Poland; 2 Department of Biology of Bacteria, Faculty of Biology and Environmental Protection, University of Lodz, ul. Banacha 12/16, 90-237 Łódź, Poland

Correction to: *Scientific Reports* 10.1038/s41598-019-53359-z, published online 19 November 2019

In the original PDF version of this Article, the Data Availability statement was obscured by the publication date. This has now been corrected in the PDF version of this Article.

In addition, there was a typographical error in the Results section under subheading ‘Influence of LPS on the nucleation and growth of PCaAP’,

“The interaction between LPS and precipitated PCaAP can take place by means of vander Waals forces or hydrogen bonds.”

now reads:

“The interaction between LPS and precipitated PCaAP can take place by means of van der Waals forces or hydrogen bonds.”

This has now been corrected in the HTML and PDF versions of this article.

